# Coffee's effects on cardiac arrhythmias, physical activity, sleep and serum glucose: Insights from the Coffee and Real‐time Atrial and Ventricular Ectopy trial

**DOI:** 10.1002/ctm2.1348

**Published:** 2023-07-27

**Authors:** Gregory M. Marcus

**Affiliations:** ^1^ Department of Medicine Division of Cardiology University of California San Francisco California USA

1

Coffee is one of the most commonly consumed beverages in the world, and yet the majority of research investigating the health effects have been limited to either observational studies prone to confounding or small laboratory‐based studies in artificial settings focused on single doses of caffeine. Despite the conventional wisdom that coffee increases the risk of arrhythmias, recent observational studies have failed to reveal a heightened risk of arrhythmias among coffee drinkers.^1^, ^2^ Caffeinated coffee notoriously disrupts sleep, but randomized studies in natural environments (i.e. participants asleep in their own beds) have been lacking. And finally, large epidemiologic studies have revealed lower mortality among coffee drinkers.^3^ This favourable prognosis is often attributed to the possibility that caffeinated coffee might motivate more physical activity or related to the observation that coffee drinkers appear to experience a lower risk of diabetes.^4^

We sought to leverage modern technology, including wearable sensors and the nearly ubiquitous use of internet‐connected smartphones, to conduct the first ambulatory randomized trial of caffeinated coffee in order to investigate near‐term effects on common arrhythmias, physical activity, sleep, serum glucose in the Coffee and Real‐time Atrial and Ventricular Ectopy (CRAVE) trial.^5^

CRAVE was a randomized, case‐crossover trial. We estimated having sufficient power to detect clinically relevant outcomes with only 100 participants. As a case‐crossover randomized trial, we had more than a thousand different outcome assessments over the 14‐day study period. Power was further enhanced by employing continuous outcomes derived from every participant.

Participants were fitted with a continuously‐recording ECG device (iRhythm), a wrist‐worn Fitbit to record step counts and sleep durations, and a continuous glucose monitor (Dexcom). They downloaded the Eureka mobile application (University of California, San Francisco) for geolocation tracking. DNA was collected and genotyped for common caffeine metabolism‐related variants.

Participants were randomly instructed to consume or avoid caffeinated coffee via daily text messages for 14 days. Adherence to their assignment was assessed in four ways: they were instructed to push a button on the ECG monitor that time‐stamped every coffee drink (a previously validated approach, which mitigates against recall bias^6^); daily surveys; reimbursements for date‐stamped receipts for coffee purchases; and geofencing for coffee shop visits. Each of these methods demonstrated that the great majority of participants followed their randomization assignment the majority of the time.

Participants were on average 39 ± 13 years old, 51% were women, and 51% were non‐Hispanic White.

Our main arrhythmia outcomes included premature atrial contractions (PACs) and premature ventricular contractions (PVCs). Recent evidence has demonstrated that the frequency of each is clinically important: more PACs predict atrial fibrillation^7^ and more PVCs predict heart failure.^8^ In both intention‐to‐treat and as‐treated analyses and consistent with prior investigations, we found no relationship between coffee consumption and PAC counts. In contrast, random assignment to coffee was associated with about 50% more PVCs, and those consuming more than 1 drink of coffee had a doubling of their PVCs (both statistically significant). Although this discordance between atrial and ventricular ectopy might appear counter‐intuitive, it fits with previous evidence. In the UK Biobank, more coffee consumption was associated with significantly less atrial arrhythmias, but, although not statistically significant, more PVCs.^2^ One of the few published studies to demonstrate significantly more arrhythmias associated with coffee consumption focused on PVCs.^9^ These data further support the notion that fundamental differences in the causes of PACs versus PVCs exist.

**FIGURE 1:**
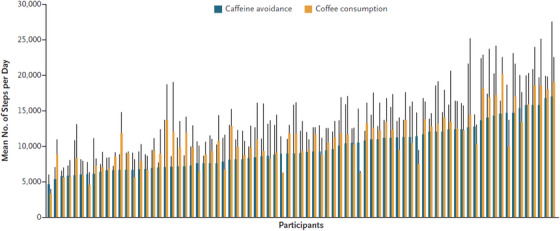
Daily step count for each participant on days of coffee consumption and days of caffeine avoidance. The x‐axis pairs each participant with their mean step counts on days randomized to avoid caffeine (blue) versus those on days randomized to consume coffee(orange) side‐by‐side. The vertical black bars indicate standard deviations. Reprinted with permission.

Random assignment to consume coffee was associated with 1,058 more steps per day (95% confidence interval [CI] 441–1675 steps, *p* = .0010, Figure 1). Every additional coffee drink was associated with 587 more steps per day (95% CI 355–820 steps, *p* < .001). This magnitude of step count differences has been associated with improved health outcomes, including reduced mortality.^10^ Presumably, the caffeine helped with the psychological motivation to exercise or enhanced physical performance in a way that made such activity easier or more enjoyable.

**FIGURE 2:**
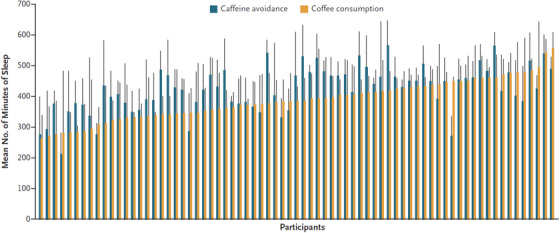
Minutes asleep for each participant after coffee consumption and caffeine avoidance. The x‐axis pairs each participant with their mean sleep durations on days randomized to avoid caffeine (blue) versus those on days randomized to consume coffee(orange) side‐by‐side. The vertical black bars indicate standard deviations. Reprinted with permission.

When randomized to coffee, participants slept about a half‐hour less per night (36 fewer minutes, 95% CI 25 to −47, *p* < .001, Figure 2), and every additional coffee drink was associated with 14 min less sleep per night (95% CI 10−18 fewer minutes, *p* < .001). Coffee notoriously disrupts sleep, but to our knowledge, the relationship had not previously been characterized, nor quantified, among ambulatory patients in a randomized trial. Of interest, there was a statistically significant interaction with caffeine metabolism‐related genetic variants: slower caffeine metabolizers experienced almost an hour less sleep on average, whereas the fastest caffeine metabolizers had no detectable difference in sleep duration when exposed to coffee. Taken together, at least two clinically relevant lessons can be drawn. First, for those seeking any opportunity to improve their sleep, these data provide rigorous evidence of a causal relationship and should bolster earnest trials to meaningfully reduce if not completely abstain from coffee to determine if it helps. Second, when a patient reports that coffee does not affect their sleep, these data demonstrate this is indeed possible.

Finally, no differences in daily mean serum glucose related to coffee consumption were observed. This suggests that prior reports that coffee reduces the risk of diabetes^4^ may be due to confounding or more chronic effects, such as related to enhanced physical activity or antioxidant properties.

It is difficult to sum up the findings of CRAVE in a single conclusion precisely because coffee has a myriad of clinically relevant health effects. Even further, these effects exhibit heterogeneity across individuals, particularly regarding sleep. The effects of coffee are relevant to basic and universal physiologic processes, and we hope these data might inform both clinicians and the lay public to help guide the consumption of this common substance based on individual‐level propensities and goals of care.
